# Preliminary Findings on the Predictive Value of Hematologic Inflammatory Indices for Survival in Treatment-Naïve Non-Metastatic Nasopharyngeal Carcinoma: A Retrospective Cohort Study

**DOI:** 10.3390/jcm15124760

**Published:** 2026-06-18

**Authors:** Muhammed Ali Coşkuner, Gökhan Köker, Gizem Zorlu Görgülügil, Gülhan Özçelik Köker, Bilgin Bahadır Başgöz, Asım Armağan Aydın, Mustafa Yıldız

**Affiliations:** 1 Department of Internal Medicine, Antalya City Hospital, 07080 Antalya, Turkey; bbbasgoz@gmail.com; 2 Department of Internal Medicine, Antalya Training and Research Hospital, 07100 Antalya, Turkey; gkhnkkr@gmail.com (G.K.); gizemzorlug@gmail.com (G.Z.G.); 3 Department of Medical Oncology, Faculty of Medicine, Akdeniz University, 07070 Antalya, Turkey; dr.gulhan.ozcelik@gmail.com; 4 Department of Medical Oncology, Antalya Training and Research Hospital, 07100 Antalya, Turkey; drarmaganaydin@gmail.com (A.A.A.); mustafa.yildiz@sbu.edu.tr (M.Y.)

**Keywords:** nasopharyngeal neoplasms, inflammation, prognosis, survival analysis

## Abstract

**Background/Objectives**: Prognostic stratification in non-metastatic nasopharyngeal carcinoma (NPC) remains challenging, particularly among patients within the same TNM stage. Readily available hematologic inflammatory indices may reflect host–tumor interactions and provide additional prognostic information beyond conventional clinicopathologic factors. This study evaluated the prognostic value of pretreatment hematologic inflammatory indices for overall survival (OS) and progression-free survival (PFS) in patients with non-metastatic NPC. **Methods**: This single-center retrospective cohort study included adult patients with non-metastatic NPC diagnosed at a tertiary referral center between 20 February 2014 and 2 May 2023, with outcomes ascertained through 12 December 2023. Pretreatment complete blood count and biochemical parameters were used to calculate the neutrophil-to-lymphocyte ratio, platelet-to-lymphocyte ratio, systemic immune-inflammation index, pan-immune-inflammation value (PIV), and hemoglobin–albumin–lymphocyte–platelet score. Receiver operating characteristic analysis determined optimal cut-off values for mortality discrimination. Associations with OS and PFS were assessed using Cox regression models. **Results**: Forty-six patients were analyzed, including 37 males. Median OS and PFS were 45.90 and 37.05 months, respectively. Compared with survivors, non-survivors were older and had lower hemoglobin and albumin levels, higher PIV, NLR, PLR, and SII values, and lower HALP scores. Although NLR showed the highest conventional ROC performance for mortality discrimination, PIV retained prognostic significance in multivariable Cox models and showed stable time-dependent discrimination for PFS. **Conclusions:** These preliminary findings suggest that pretreatment inflammatory indices, particularly composite markers such as PIV, may provide adjunctive prognostic information in treatment-naïve non-metastatic NPC, pending larger prospective validation.

## 1. Introduction

Nasopharyngeal carcinoma (NPC) is a biologically and epidemiologically distinct head and neck malignancy characterized by striking geographic and ethnic disparities. Although NPC is rare in most regions, it remains endemic in parts of East and Southeast Asia, North Africa, and the Arctic, where population-level risk is substantially higher than in low-incidence settings [1,2]. Globally, GLOBOCAN 2022 estimates indicate approximately 120,434 new cases of NPC and approximately 73,482 deaths, underscoring a persistent mortality burden despite advances in diagnosis and treatment [3]. Contemporary global modeling suggests that, driven by population growth and aging, the absolute number of cases and deaths is likely to continue rising over the coming decades unless meaningful reductions in incidence and mortality are achieved [4]. Despite refinements in radiotherapy and systemic regimens, relapse remains common in high-risk disease, and although immune checkpoint inhibitors have expanded options in recurrent/metastatic NPC, benefits are heterogeneous—highlighting the need for robust baseline risk stratification to inform surveillance and treatment personalization [5].

The etiopathogenesis of NPC is multifactorial. It reflects a complex interaction between viral, host genetic, and environmental factors. Epstein–Barr virus (EBV) infection is central for NPC oncogenesis, especially in endemic non-keratinizing subtypes. EBV-related biomarkers have increasingly informed screening and prognostication strategies in high-incidence regions [6]. Alongside EBV, hereditary susceptibility and lifestyle or dietary exposures contribute to risk heterogeneity. Epidemiologic syntheses highlight the roles of genetic predisposition and environmental carcinogens including traditional dietary patterns. Tobacco and alcohol may also contribute, particularly in selected disease contexts [1,2,6]. Clinically, NPC is often diagnosed in locally advanced stages because early symptoms can be nonspecific. Even within non-metastatic disease, outcomes vary widely, such as tumor burden, nodal involvement, treatment response, and patient-related factors [1].

Risk stratification in non-metastatic NPC still relies primarily on anatomical staging systems; however, substantial outcome variability persists among patients within the same tumor–node–metastasis (TNM) stage [1]. EBV DNA and other molecular markers can improve prognostic precision, but they may be unavailable, costly, or inconsistently standardized across centers [1,6]. Therefore, there is ongoing interest in simple, low-cost, routinely accessible biomarkers that reflect host–tumor interactions and can complement conventional staging to refine prognosis and inform personalized surveillance or treatment intensification strategies.

In this context, systemic inflammation-based hematologic inflammatory indices derived from complete blood count parameters and basic biochemical findings have gained traction across oncology. Accumulating evidence suggests that cancer-related inflammation contributes to tumor growth, angiogenesis, invasion, metastatic dissemination, and immune evasion, while peripheral blood cellular profiles provide a practical window into the balance between protumor inflammatory activity, antitumor immune competence, and immunonutritional reserve. This concept is particularly relevant in NPC, a malignancy closely linked to EBV-associated oncogenesis, immune dysregulation, and marked heterogeneity in treatment response and long-term outcomes. Among routinely available indices, NLR reflects the balance between neutrophil-driven systemic inflammation and lymphocyte-mediated antitumor immune surveillance, whereas PLR incorporates the potential contribution of platelets to tumor progression, angiogenesis, circulating tumor cell protection, and immune escape. In NPC specifically, pretreatment NLR and PLR have been repeatedly associated with survival endpoints, and recent quantitative syntheses reinforce their prognostic relevance [7,8]. Moreover, combined approaches—such as simultaneous risk grouping using both pretreatment NLR and PLR—have been proposed to enhance prognostic discrimination in non-metastatic NPC cohorts [7].

Beyond single ratios such as NLR and PLR, composite inflammation-based indices have also been explored to better capture the systemic tumor–host interplay in oncology. In NPC, the systemic immune-inflammation index (SII) has shown prognostic associations in observational cohorts and has been supported by systematic reviews and meta-analyses [9,10]. Its relevance is also supported beyond NPC; a meta-analysis in head and neck cancers reported that elevated pretreatment SII was associated with more advanced tumor and nodal status and poorer survival outcomes [11]. The pan-immune-inflammation value (PIV) has also been investigated in NPC, and higher pretreatment PIV has generally been associated with inferior survival [12,13]. Beyond NPC, PIV and related systemic inflammation-based indices have shown prognostic potential across solid tumors, further supporting their role as integrative markers of systemic immune–inflammatory status [14,15]. In parallel, immunonutritional indices such as the hemoglobin–albumin–lymphocyte–platelet (HALP) score have also been evaluated in NPC, suggesting potential prognostic utility in selected non-metastatic subgroups [16].

However, effect sizes and optimal cut-offs vary across studies, and many reports evaluate single indices, limiting head-to-head comparison and pragmatic implementation in routine care. Accordingly, we evaluated whether pretreatment hematologic inflammatory indices derived from routinely obtained laboratory tests were associated with OS and PFS in patients with non-metastatic NPC and assessed which indices retained prognostic significance alongside established clinicopathological variables. We hypothesized that an unfavorable pretreatment systemic inflammatory profile, characterized by higher inflammation-based indices and lower immunonutritional reserve, would be associated with poorer OS and PFS. We further hypothesized that composite indices integrating multiple hematologic components may provide additional prognostic information beyond individual cell-based ratios.

## 2. Materials and Methods

### 2.1. Aim, Study Design and Setting

The aim of this study was to evaluate the prognostic value of pretreatment hematologic inflammatory indices, including NLR, PLR, SII, PIV, and HALP, for OS and PFS in patients with treatment-naïve non-metastatic nasopharyngeal carcinoma. This single-center, retrospective cohort study included adult patients with non-metastatic NPC who were diagnosed between 20 February 2014 and 2 May 2023 and followed at the oncology center of a tertiary care hospital, with outcomes ascertained through 12 December 2023. Clinical data were retrieved retrospectively from the hospital electronic medical record system and archived patient files.

### 2.2. Study Population

Eligible participants were adult patients diagnosed with treatment-naïve non-metastatic NPC during the study period who had sufficient demographic, clinicopathological, treatment-related, laboratory, and follow-up data available in the medical records to calculate hematologic inflammatory indices and perform survival analyses.

**Figure 1:**
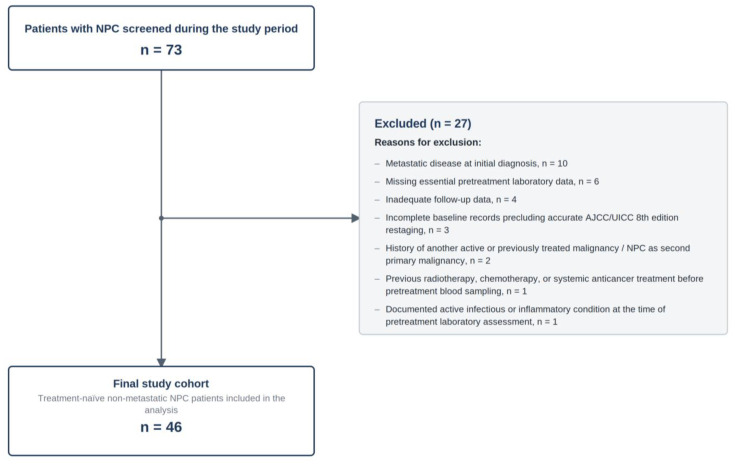
STROBE-compliant patient selection flow diagram of the study cohort.

Patients were excluded if they had metastatic disease at initial diagnosis; missing essential pretreatment laboratory data precluding index calculation; missing follow-up data required to determine OS and/or PFS; a history of another active or previously treated malignancy; NPC as a second primary malignancy; or previous radiotherapy, chemotherapy, or systemic anticancer treatment before the pretreatment blood sampling used for index calculation. To minimize potential confounding of hematologic inflammatory indices, patients with documented active infectious or inflammatory conditions at the time of pretreatment laboratory assessment, including acute upper respiratory tract infection, active sinusitis, dental infection, skin or soft-tissue infection, or other clinically evident inflammatory disorders, were also excluded. The patient screening, exclusion process, and final cohort derivation are summarized in a STROBE-compliant flow diagram (Figure 1).

### 2.3. Data Collection and Variables

For each patient, the following data were extracted:

Demographic variables: age at diagnosis and sex.

Clinicopathological variables: tumor and nodal status (TNM), overall stage, comorbidities, and EBV DNA.

Treatment variables: induction chemotherapy (yes/no) and other treatment details recorded during routine care. All patients were managed according to institutional standard-of-care protocols, typically including definitive concurrent chemoradiotherapy (CCRT) with induction chemotherapy at the clinician’s discretion in selected patients.

Laboratory variables: routinely obtained pretreatment complete blood count and biochemistry measurements used to derive inflammatory indices (including neutrophils, lymphocytes, monocytes, platelets, hemoglobin, albumin).

All laboratory variables were obtained prior to administration of anticancer treatment (pretreatment), based on routinely collected measurements during the diagnostic/treatment planning period. Tumor staging was performed according to the 8th edition of the TNM classification system of the American Joint Committee on Cancer (AJCC) and the Union for International Cancer Control (UICC). Because the study period included patients diagnosed before the implementation of the AJCC/UICC 8th edition, all available baseline clinical, endoscopic, radiologic, and pathological records were retrospectively reviewed and harmonized according to AJCC/UICC 8th edition criteria. For patients diagnosed in earlier years, original staging information was not used directly unless sufficient source data were available to confirm the corresponding 8th edition T, N, and stage group classifications. Patients with insufficient baseline information for accurate retrospective restaging were excluded from the analysis. Baseline plasma EBV DNA was measured at diagnosis using a quantitative PCR assay in the institutional laboratory and categorized as <ULN versus ≥ULN according to the laboratory reference range. EBV DNA was evaluated because of its established clinical and prognostic relevance in NPC. HPV status and other viral or molecular markers were not routinely assessed as part of the institutional diagnostic workflow during the study period and were therefore not included in the present analysis.

#### Hematologic Inflammatory Indices

Pretreatment inflammatory indices were calculated according to the following standard formulas:

NLR = Neutrophils (10^9^/L)/Lymphocytes (10^9^/L)

PLR = Platelets (10^9^/L)/Lymphocytes (10^9^/L)

SII = (Platelets (10^9^/L) × Neutrophils (10^9^/L))/Lymphocytes (10^9^/L)

PIV = (Platelets (10^9^/L) × Neutrophils (10^9^/L) × Monocytes (10^9^/L))/Lymphocytes (10^9^/L)

HALP = (Hemoglobin (g/L) × Albumin (g/L) × Lymphocytes (10^9^/L))/Platelets (10^9^/L)

### 2.4. Outcomes and Follow-Up

The primary endpoints were OS and PFS. OS was defined as the time from the date of diagnosis to death from any cause or the date of last documented follow-up. PFS was defined as the time from the date of diagnosis to the first documented disease progression (including locoregional recurrence and/or distant metastasis) or death from any cause, whichever occurred first. Progression/recurrence was ascertained during routine follow-up based on radiologic evaluation (contrast-enhanced computed tomography, magnetic resonance imaging, and/or positron emission tomography–computed tomography) and/or clinical documentation in the medical records, with histopathologic confirmation when clinically feasible. Patients were accrued between 20 February 2014 and 2 May 2023. Follow-up information was collected through 12 December 2023 (database lock/administrative censoring date). Patients without an event were censored at the date of their last documented disease assessment occurring on or before 12 December 2023.

### 2.5. Ethical Considerations

The study protocol was reviewed and approved by the local ethics committee (Approval date: 22 January 2026; Decision number: 2/17). Due to the retrospective study design and the use of anonymized routinely collected clinical data, the requirement for written informed consent was waived by the local ethics committee in accordance with institutional policies and applicable local regulations.

### 2.6. Statistical Analysis

Statistical analyses were performed using IBM SPSS Statistics software (version 27.0; IBM Corp., Armonk, NY, USA) and R software (version 4.4.3; R Foundation for Statistical Computing, Vienna, Austria). Continuous variables were assessed for normality using the Shapiro–Wilk test. Normally distributed variables are presented as mean ± standard deviation (SD) and were compared using Student’s *t*-test, whereas non-normally distributed variables are reported as median and interquartile range (IQR) and were compared using the Mann–Whitney *U* test. Categorical variables are presented as frequencies and percentages and were compared using the chi-square test or Fisher’s exact test, as appropriate.

The median follow-up duration was estimated using the reverse Kaplan–Meier method. Receiver operating characteristic (ROC) curve analyses were performed to evaluate the discriminatory performance of hematologic inflammatory indices for mortality. Area under the curve (AUC) values, optimal cut-off points determined by the Youden index, sensitivity, specificity, and corresponding 95% confidence intervals (CIs) were calculated.

To assess the potential optimism associated with deriving and evaluating ROC-based cut-offs within the same cohort, an internal validation procedure was performed using 1000 bootstrap resamples. Bootstrap-estimated AUC distributions and corresponding 95% confidence intervals were calculated for each inflammatory index. The bootstrap analysis was used to evaluate the stability and robustness of the observed discrimination performance across repeated resampling iterations.

Because conventional ROC analysis does not account for censoring in survival data, time-dependent ROC analyses were additionally performed using an inverse probability of censoring weighting (IPCW) approach. Time-specific AUC values [AUC(t)] were calculated for overall survival (OS) and progression-free survival (PFS) at predefined follow-up time points (12, 24, 36, and 60 months).

Point-biserial correlation coefficients were calculated to assess the associations between inflammatory indices and mortality status. The effects of clinicopathological variables and inflammatory indices on OS and PFS were evaluated using Cox proportional hazards regression models. Variables were initially assessed in univariate Cox regression analyses. Age, sex, and variables demonstrating statistical significance in univariate analyses were subsequently entered into multivariable Cox regression models.

Prior to multivariable modeling, multicollinearity among candidate covariates was assessed using correlation matrices and variance inflation factor (VIF) statistics. Variables demonstrating substantial collinearity (correlation coefficient > 0.70 or VIF > 3) were not entered simultaneously into the same multivariable model. Given the mathematical overlap among the evaluated inflammatory indices, separate multivariable models were constructed to avoid instability arising from collinearity.

All statistical tests were two-sided, and a *p*-value < 0.05 was considered statistically significant. Results are reported with 95% confidence intervals where applicable.

## 3. Results

A total of 46 adult patients with treatment-naïve non-metastatic NPC were included, and 33 (71.7%) were alive at the last follow-up. The median follow-up was 75.7 months (IQR 37.0–84.0 months), ranging from 7.4 to 117.7 months. Baseline demographic and clinical characteristics, EBV DNA status, laboratory parameters, and inflammatory indices are summarized in Table 1, and disease distribution with treatment characteristics are presented in Table 2.

When stratified by vital status, non-survivors were older and exhibited a less favorable baseline biochemical profile, including lower hemoglobin and albumin levels (Table 1). Hematologic inflammatory indices also differed between survivors and non-survivors, with higher PIV, NLR, PLR, and SII and lower HALP among non-survivors (Table 1).

**Figure 2:**
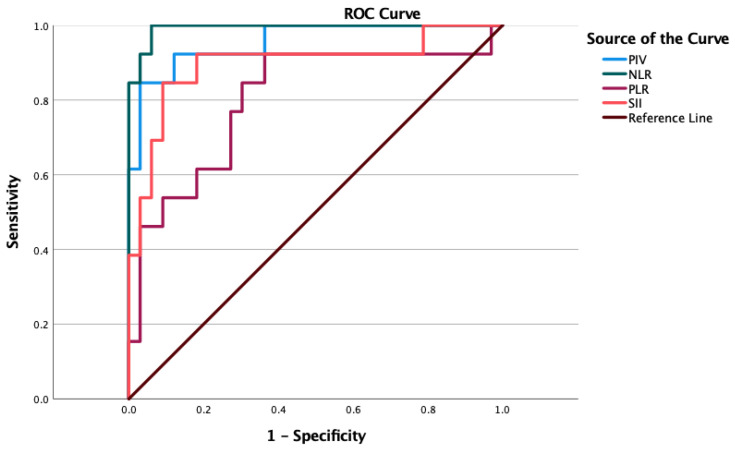
Receiver operating characteristic (ROC) curves of PIV, NLR, PLR, and SII for predicting patient mortality. ROC: receiver operating characteristic; PIV, pan-immune-inflammation value; NLR, neutrophil-to-lymphocyte ratio; PLR, platelet-to-lymphocyte ratio; SII, systemic immune-inflammation index.

**Figure 3:**
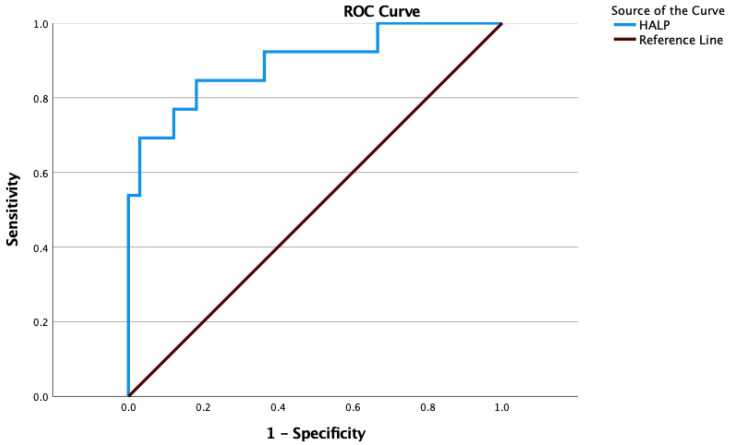
Receiver operating characteristic (ROC) curve of HALP for predicting patient mortality. ROC: receiver operating characteristic; HALP, hemoglobin–albumin–lymphocyte–platelet score.

**Figure 4:**
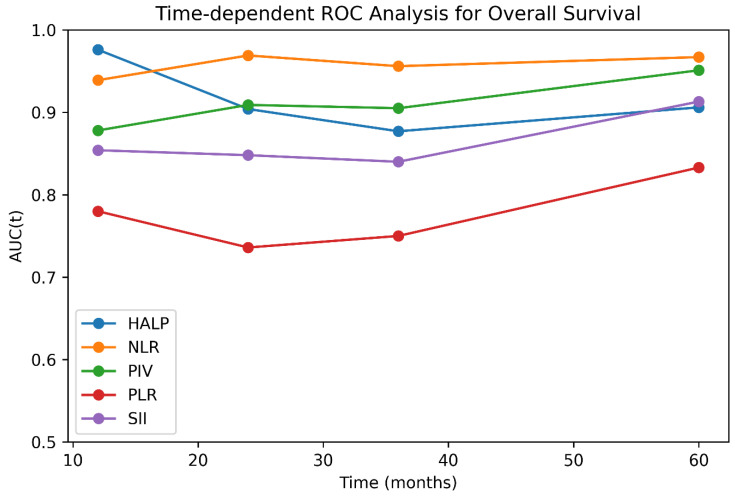
Time-dependent ROC curves for overall survival. ROC, receiver operating characteristic; HALP, hemoglobin–albumin–lymphocyte–platelet score; PIV, pan-immune-inflammation value; NLR, neutrophil-to-lymphocyte ratio; PLR, platelet-to-lymphocyte ratio; SII, systemic immune-inflammation index.

**Figure 5:**
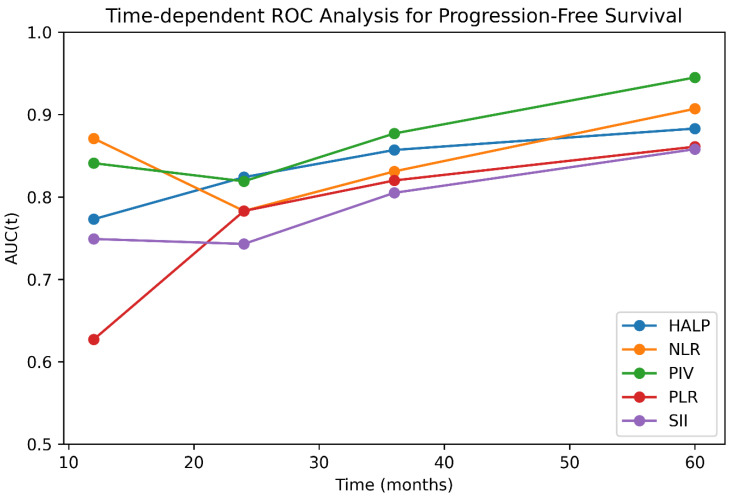
Time-dependent ROC curves for progression-free survival. ROC: receiver operating characteristic; HALP, hemoglobin–albumin–lymphocyte–platelet score; PIV, pan-immune-inflammation value; NLR, neutrophil-to-lymphocyte ratio; PLR, platelet-to-lymphocyte ratio; SII, systemic immune-inflammation index.

For the overall cohort, the median OS was 45.9 months and the median PFS was 37.1 months (Table 1). Point-biserial correlation analyses demonstrated significant associations between inflammatory indices and mortality (Table 3): PIV, NLR, PLR, and SII correlated positively with non-survival, whereas HALP showed an inverse correlation. ROC analyses further supported the discriminative ability of these indices for non-survival, with ROC curves presented in Figure 2 (PIV, NLR, PLR, SII) and Figure 3 (HALP). Among the indices, NLR demonstrated the highest discrimination (AUC 0.993), followed by PIV (AUC 0.956); complete ROC-derived cut-offs, AUCs with 95% CIs, and sensitivity/specificity estimates are provided in Table 3. To appropriately account for right-censored survival data, time-dependent ROC analyses were conducted for OS and PFS at 12, 24, 36, and 60 months using inverse probability of censoring weighting (Figure 4 and Figure 5) (Table 4). For OS, AUC(t) values remained consistently high across indices, with NLR, PIV and HALP demonstrating the strongest and most stable time-varying discrimination, whereas PLR showed comparatively lower discriminative performance. For PFS, PIV and NLR exhibited robust discrimination across time, with higher AUC(t) values observed at later time points, while PLR and SII demonstrated moderate but gradually improving performance.

To assess the potential optimism associated with deriving and testing ROC-based cutoffs within the same cohort, an internal validation analysis was performed using 1000 bootstrap resamples. The discriminative performance of the evaluated inflammatory indices remained broadly consistent across bootstrap iterations. For mortality prediction, bootstrap-estimated AUCs (95% confidence intervals) were 0.998 (0.986–1.000) for NLR, 0.967 (0.903–1.000) for PIV, 0.918 (0.783–0.998) for SII, 0.893 (0.758–0.990) for HALP, and 0.802 (0.625–0.942) for PLR. These findings demonstrated stable discrimination across repeated resampling procedures and supported the robustness of the primary ROC analyses (Table 5).

In univariate Cox regression for OS, age (Hazard Ratio [HR] 1.051) and EBV DNA ≥ upper limit of normal (ULN) (HR 5.188) were significantly associated with mortality (Table 6). All inflammatory indices were significantly associated with OS both as continuous measures and when dichotomized using ROC-derived thresholds (Table 6). In multivariate analysis, the model including continuous PIV identified PIV (HR 1.001) and EBV DNA ≥ ULN (HR 6.716) as independent predictors of OS (Table 7). In the alternative model using dichotomized PIV, PIV above the ROC-derived cut-off remained independently associated with higher mortality (HR 36.452), whereas EBV DNA ≥ ULN did not retain statistical significance (Table 7).

For PFS, EBV DNA ≥ ULN was significantly associated with poorer outcomes in univariate analysis (HR 4.183; Table 8). All inflammatory indices were also significantly associated with PFS as continuous and dichotomized variables (Table 8). In multivariate Cox regression, the continuous PIV model retained independent associations for PIV (HR 1.001) and EBV DNA ≥ ULN (HR 3.528) (Table 9). In the dichotomized model, PIV above the ROC-derived cut-off remained independently associated with poorer PFS (HR 9.525), while EBV DNA ≥ ULN was not statistically significant (Table 9).

## 4. Discussion

In this single-center retrospective cohort of patients with treatment-naïve non-metastatic NPC, five key findings emerged. First, pretreatment hematologic inflammatory indices were significantly associated with both OS and PFS. Second, patients with poorer outcomes exhibited an unfavorable systemic inflammatory and immunonutritional profile, characterized by higher PIV, NLR, PLR, and SII values and lower HALP scores. Third, among the evaluated markers, PIV retained independent prognostic significance in multivariable models, whereas other indices were not entered into the same models because of collinearity with PIV. Fourth, baseline EBV DNA status remained clinically relevant and was associated with survival outcomes. Fifth, these findings suggest that routinely available blood-based indices may provide practical and low-cost prognostic information for risk stratification in NPC, although the results should be interpreted as preliminary and hypothesis-generating. Overall, these findings support the concept that composite inflammation scores derived from routinely available blood counts may aid prognostic assessment in NPC [13,17,18].

Systemic inflammation is increasingly recognized as a critical component of tumor progression, influencing angiogenesis, invasion, and immune evasion, while peripheral blood cell profiles offer a pragmatic surrogate of the host–tumor immune balance [17]. In NPC, this concept has translated into extensive work on CBC-derived indices. NLR and PLR are the most widely studied, and meta-analytic evidence has consistently linked elevated pretreatment NLR and PLR with inferior survival outcomes across NPC cohorts [8,19]. Similarly, SII, which integrates platelets, neutrophils, and lymphocytes, has demonstrated prognostic relevance and has been supported by systematic reviews/meta-analyses in NPC [10]. In our cohort, NLR, PLR, and SII were all significantly associated with OS and PFS in univariable analyses, aligning with the direction and magnitude of effects reported in the broader literature [8,10,19].

A key aspect of our analysis is that PIV was prioritized for multivariable modeling because the other indices displayed collinearity with PIV. This is biologically and mathematically plausible: PIV includes neutrophils, platelets, monocytes, and lymphocytes, thereby capturing overlapping information contained in NLR, PLR, and SII. Although conventional ROC analysis yielded a slightly higher AUC for NLR than for PIV in mortality discrimination, PIV was prioritized for multivariable survival modeling for two main reasons. First, the inflammatory indices were mathematically and statistically interrelated, and collinearity diagnostics indicated that NLR, SII, and PIV should not be entered simultaneously into the same Cox model. PIV was therefore selected as a comprehensive and parsimonious composite index because it integrates monocytes in addition to neutrophils, platelets, and lymphocytes. Second, conventional ROC analysis evaluates discrimination for a fixed binary outcome and does not account for right-censoring over time. In the time-dependent ROC analysis for PFS, PIV showed stable longitudinal discrimination and yielded higher AUC(t) values than NLR at 24, 36, and 60 months (0.819, 0.877, and 0.945, respectively), compared with NLR (0.783, 0.831, and 0.907, respectively). These findings support the use of PIV as a representative composite inflammatory index in the multivariable models, while NLR and the other indices were fully reported in univariable, conventional ROC, and time-dependent ROC analyses. In parallel with our findings, emerging NPC-specific studies have reported that higher pretreatment PIV is associated with worse survival endpoints, suggesting that PIV may be a promising CBC-derived marker in this disease [13]. Moreover, broader oncology evidence also supports the prognostic role of PIV across multiple solid tumors and treatment contexts [18].

Although HALP did not enter our multivariable models, it showed a clear univariable signal in our cohort, with lower pretreatment HALP values observed in non-survivors and significant associations with OS and PFS. This finding is consistent with the biological premise that immunonutritional reserve—reflected by hemoglobin and albumin together with lymphocyte and platelet components—may influence tolerance to therapy, susceptibility to complications, and long-term outcomes. In NPC, HALP has been reported as an independent prognostic factor and has been incorporated into prognostic nomograms in selected non-metastatic subgroups, supporting its potential clinical relevance [16]. In our study, HALP was not modeled concurrently with PIV and other indices because these metrics are mathematically interdependent and showed collinearity; therefore, our results should be interpreted as identifying the most informative marker within this dataset rather than the incremental value of combining multiple indices [20].

We also observed that baseline EBV DNA ≥ ULN was associated with survival outcomes and retained independent significance in at least one of the multivariable frameworks, which is consistent with the well-established prognostic role of pretreatment plasma EBV DNA in NPC [21]. Meta-analytic data indicate that higher pretreatment EBV DNA correlates with increased risks of death and disease progression in newly diagnosed NPC [21]. At the same time, EBV DNA quantification can be affected by pre-analytical and analytical variability, and assay harmonization has been emphasized as a prerequisite for consistent biomarker-guided applications across centers [22]. Within this context, CBC-based indices such as PIV may be particularly attractive as complementary tools because they are inexpensive, universally accessible, and easily calculated from routine pretreatment laboratory parameters [13,18].

In our cohort, induction chemotherapy was not significantly associated with OS or PFS in univariable analyses. This should be interpreted cautiously given the limited sample size and the likelihood of confounding by indication in retrospective datasets, particularly because the induction-chemotherapy subgroup was small and the number of events was limited, which may have precluded a mature estimate of treatment effect. In contemporary randomized evidence, induction chemotherapy—particularly gemcitabine plus cisplatin before concurrent chemoradiotherapy—has improved PFS and OS in locoregionally advanced NPC compared with chemoradiotherapy alone [23]. Therefore, the absence of a statistically significant benefit in our analysis more likely reflects restricted power, treatment-selection bias, and heterogeneity in stage and risk profiles rather than a true lack of effect, particularly given the small induction-chemotherapy subgroup in our cohort [23].

From a clinical perspective, these findings should not be interpreted as sufficient to guide treatment escalation or de-escalation on their own. However, pretreatment inflammatory indices may have potential value as adjunctive tools for preliminary risk stratification when interpreted together with TNM stage, EBV DNA status, treatment characteristics, and patient-related factors. Patients with an unfavorable inflammatory profile may warrant closer post-treatment surveillance, more careful clinical counseling regarding recurrence or progression risk, and consideration for inclusion in future prospective studies evaluating risk-adapted follow-up or treatment strategies. Because these indices are inexpensive, routinely available, and easily calculated from standard pretreatment laboratory tests, they may be particularly useful in settings where advanced molecular or imaging-based prognostic tools are not readily accessible. Nevertheless, clinical implementation requires external validation and prospective assessment before these markers can be incorporated into decision-making algorithms.

Several limitations merit consideration. First, the retrospective, single-center design may introduce selection bias and limits generalizability. Second, the cohort size was modest and the number of events was limited, which reduces statistical power, increases the risk of overfitting and model instability, and restricts the number of covariates that can be reliably included in multivariable Cox regression models. For this reason, multivariable models were constructed parsimoniously, and the findings should be interpreted as exploratory and hypothesis-generating rather than definitive predictive models. Although the use of survival-based analyses, collinearity assessment, and time-dependent ROC analysis accounting for right-censoring strengthened the analytical framework, external validation is required before clinical implementation. Third, ROC-derived cut-offs were determined within the same dataset used for outcome modeling; therefore, these data-driven thresholds may overestimate apparent discrimination and should not be considered clinically actionable without validation. Fourth, NPC stage is a major determinant of prognosis and may influence the relationship between systemic inflammatory indices and survival outcomes. Although T stage, N stage, stage group, treatment modality, and comorbidity status were evaluated in univariable analyses, they were not significantly associated with OS or PFS in this modest cohort and were therefore not included in the final multivariable models to reduce the risk of overfitting. However, the absence of statistically significant associations in univariable analyses may partly reflect limited statistical power rather than a true lack of prognostic relevance. Moreover, performance status was not consistently available in the retrospective records and could not be incorporated into the models. Therefore, residual confounding related to tumor burden, nodal status, stage distribution, treatment selection, performance status, and comorbidity burden cannot be excluded. Fifth, because NPC is an endemic malignancy with region-specific etiologic and biologic features—especially EBV-associated disease patterns—our single-center findings may have limited generalizability across endemic and non-endemic settings. Finally, because PIV encompasses components of other indices, collinearity restricted the ability to directly compare the independent contributions of NLR, PLR, SII, and PIV in the same multivariable model; alternative approaches such as penalized regression, internal validation, or pre-specified biomarker selection may be informative in larger datasets.

In conclusion, pretreatment hematologic inflammatory indices were associated with OS and PFS in patients with treatment-naïve non-metastatic NPC, and PIV retained independent prognostic significance in multivariable models. These preliminary findings suggest that routinely available blood-based indices may provide practical, low-cost prognostic information when interpreted together with established clinicopathological variables and EBV DNA status. Future studies should validate these findings in larger multicenter cohorts and investigate whether additional blood-based biomarkers, including albumin-derived scores, the prognostic nutritional index, the systemic inflammation response index, the lymphocyte-to-monocyte ratio, and dynamic changes in EBV DNA, can further improve risk stratification in non-metastatic NPC.

## 5. Conclusions

Pretreatment hematologic inflammatory indices were associated with OS and PFS in patients with treatment-naïve non-metastatic NPC. Among the evaluated markers, PIV retained prognostic significance in multivariable models and may serve as a practical composite indicator of systemic immune–inflammatory status. These preliminary findings support the potential role of routinely available blood-based indices in adjunctive risk stratification, but larger prospective multicenter studies are required to validate cut-off values and determine their clinical utility.

## Figures and Tables

**Table 1:** Baseline clinicopathological characteristics and pretreatment hematologic inflammatory indices according to vital status.

Variable	Total (n = 46)	Survivors (n = 33)	Non-Survivors (n = 13)	*p*
Age (years), mean (SD)	55.04 (14.90)	51.45 (15.45)	64.15 (8.81)	<0.001
Male sex, n (%)	37 (80.4)	27 (81.8)	10 (76.9)	0.698
BMI (kg/m^2^), mean (SD)	24.77 (4.62)	24.72 (4.23)	24.91 (5.57)	0.913
Comorbidity present, n (%)	14 (30.4)	8 (24.24)	6 (46.15)	0.171
WBC (×10^3^/μL), median (IQR)	7.05 (2.25)	7.00 (2.05)	7.40 (4.65)	0.651
Hemoglobin (g/dL), median (IQR)	14.15 (2.53)	14.50 (1.95)	12.30 (3.30)	0.012
Platelets (×10^3^/μL), mean (SD)	264.20 (82.69)	274.42 (73.92)	238.23 (100.25)	0.253
Albumin (g/L), median (IQR)	44.00 (7.25)	45.00 (6.00)	40.00 (12.50)	0.047
LDH (U/L), median (IQR)	202.00 (71.00)	212.00 (64.75)	181.00 (55.00)	0.146
Uric acid (mg/dL), mean (SD)	5.34 (1.47)	5.18 (1.53)	5.71 (1.27)	0.246
CRP (mg/L), median (IQR)	6.55 (18.98)	5.00 (14.98)	16.65 (36.20)	0.182
EBV DNA ≥ ULN (copies/mL), n (%)	25 (54.3)	15 (45.4)	10 (76.9)	0.099
Indices				
PIV, median (IQR)	391.29 (642.22)	308.86 (243.70)	1255.69 (1566.42)	<0.001
NLR, median (IQR)	2.54 (2.87)	2.25 (0.79)	6.60 (4.63)	<0.001
PLR, median (IQR)	162.20 (86.20)	133.53 (73.81)	220.17 (132.83)	0.002
SII, median (IQR)	674.59 (691.18)	564.22 (348.43)	1386.79 (1605.41)	<0.001
HALP, median (IQR)	36.95 (29.02)	43.59 (21.97)	20.62 (13.12)	<0.001
Survival				
Overall survival (months), median (IQR)	45.90 (59.60)	73.50 (51.55)	23.60 (31.40)	0.004
Progression-free survival (months), median (IQR)	37.05 (65.50)	57.20 (66.95)	13.10 (20.85)	<0.001

n: number; SD: Standard deviation; BMI: Body mass index; WBC: White blood cells; IQR: Interquartile range; LDH: Lactate dehydrogenase; CRP: C-reactive protein; EBV, Epstein–Barr virus; ULN, upper limit of normal; PIV, pan-immune-inflammation value; NLR, neutrophil-to-lymphocyte ratio; PLR, platelet-to-lymphocyte ratio; SII, systemic immune-inflammation index; HALP, hemoglobin–albumin–lymphocyte–platelet score. *p* < 0.05 considered statistically significant.

**Table 2:** Clinicopathological characteristics and baseline indices of the study population.

T (n, %)	Total (n = 46)
1	9 (19.6)
2	15 (32.6)
3	9 (19.6)
4	13 (28.3)
N (n, %)	
0	3 (6.5)
1	17 (37.0)
2	24 (52.2)
3	2 (4.3)
Stage (n, %)	
II	9 (19.6)
III	23 (50.0)
IVA	14 (30.4)
Treatment (n, %)	
Definitive CCRT	38 (82.6)
Induction + CCRT	8 (17.4)
EBV DNA	
<ULN (copies/mL) (n, %)	21 (45.7)
≥ULN (copies/mL) (n, %)	25 (54.3)

T, tumor category; N, nodal category; CCRT, concurrent chemoradiotherapy; EBV, Epstein–Barr virus; ULN, upper limit of normal.

**Table 3:** Point biserial correlation and ROC analyses of patient mortality across indices.

	Point Biserial Correlation		ROC				
Index	r	*p*	Cutoff	Sensitivity (%)	Specificity (%)	AUC (95% CI)	*p*
PIV	0.711	<0.001	593.53	92.3	87.1	0.956 (0.894–1.000)	<0.001
NLR	0.769	<0.001	4.05	92.3	97.0	0.993 (0.978–1.000)	<0.001
PLR	0.471	<0.001	169.54	76.9	72.7	0.802 (0.646–0.958)	0.002
SII	0.620	<0.001	1028.63	84.6	90.9	0.897 (0.777–1.000)	<0.001
HALP	−0.613	<0.001	33.01	84.6	81.8	0.893 (0.780–1.000)	<0.001

ROC, receiver operating characteristic; r, point-biserial correlation coefficient; AUC, area under the curve; CI, confidence interval; PIV, pan-immune-inflammation value; NLR, neutrophil-to-lymphocyte ratio; PLR, platelet-to-lymphocyte ratio; SII, systemic immune-inflammation index; HALP, hemoglobin–albumin–lymphocyte–platelet score.

**Table 4:** Time-dependent AUC(t) values of pretreatment inflammatory indices for overall survival and progression-free survival.

Index	Overall Survival AUC(t)				Progression-free Survival AUC(t)			
	12 Months	24 Months	36 Months	60 Months	12 Months	24 Months	36 Months	60 Months
HALP	0.976	0.904	0.877	0.906	0.773	0.824	0.857	0.883
NLR	0.939	0.969	0.956	0.967	0.871	0.783	0.831	0.907
PIV	0.878	0.909	0.905	0.951	0.841	0.819	0.877	0.945
PLR	0.780	0.736	0.750	0.833	0.627	0.783	0.820	0.861
SII	0.854	0.848	0.840	0.913	0.749	0.743	0.805	0.858

AUC(t), time-dependent area under the curve; NLR, neutrophil-to-lymphocyte ratio; PLR, platelet-to-lymphocyte ratio; SII, systemic immune-inflammation index; PIV, pan-immune-inflammation value; HALP, hemoglobin–albumin–lymphocyte–platelet score. AUC(t) values were estimated using inverse probability of censoring weighting at prespecified time points.

**Table 5:** Internal bootstrap validation of the discriminatory performance of pretreatment inflammatory indices for mortality prediction.

Index	Original AUC	Bootstrap AUC	Bootstrap 95% CI
PIV	0.956	0.967	0.903–1.000
NLR	0.993	0.998	0.986–1.000
PLR	0.802	0.802	0.625–0.942
SII	0.897	0.918	0.783–0.998
HALP	0.893	0.893	0.758–0.990

AUC, area under the curve; CI, confidence interval; PIV, pan-immune-inflammation value; NLR, neutrophil-to-lymphocyte ratio; PLR, platelet-to-lymphocyte ratio; SII, systemic immune-inflammation index; HALP, hemoglobin–albumin–lymphocyte–platelet score. Bootstrap estimates were obtained using 1000 resampling iterations.

**Table 6:** Univariate cox regression analysis on overall survival.

	Univariate Cox Regression Analysis				
	β	HR	95% CI		*p*
			Lower	Upper	
Age	0.050	1.051	1.005	1.099	0.026
Male sex	0.087	1.091	0.298	3.994	0.896
EBV DNA ≥ ULN (copies/mL)	1.646	5.188	1.384	19.453	0.015
PIV	0.001	1.001	1.001	1.002	<0.001
PIV > Cutoff	2.899	18.158	4.011	82.200	<0.001
NLR	0.679	1.971	1.495	2.600	<0.001
NLR > Cutoff	3.828	45.973	5.930	356.438	<0.001
PLR	0.014	1.014	1.008	1.021	<0.001
PLR > Cutoff	1.900	6.686	1.822	24.539	<0.001
SII	0.002	1.002	1.001	1.002	<0.001
SII > Cutoff	2.899	18.156	4.011	82.200	<0.001
HALP	−0.141	0.869	0.803	0.940	<0.001
HALP < Cutoff	2.758	15.774	3.437	72.393	<0.001
Induction + CCRT (vs. definitive CCRT)	0.980	2.665	0.346	20.506	0.346
Stage 3–4 A (vs. stage II)	1.056	2.874	0.374	22.108	0.311
Comorbidity present	0.782	2.186	0.729	6.560	0.163

β, regression coefficient; HR, hazard ratio; CI, confidence interval; EBV, Epstein–Barr virus; ULN, upper limit of normal; PIV, pan-immune-inflammation value; NLR, neutrophil-to-lymphocyte ratio; PLR, platelet-to-lymphocyte ratio; SII, systemic immune-inflammation index; HALP, hemoglobin–albumin–lymphocyte–platelet score; CCRT, concurrent chemoradiotherapy. *p* < 0.05 considered statistically significant.

**Table 7:** Multivariate cox regression analysis on overall survival.

	Multivariate Cox Regression Analysis Model 1 (Continuous PIV)					Multivariate Cox Regression Analysis Model 2 (PIV Dichotomized by Cutoff)				
	β	HR	95% CI		*p*	β	HR	95% CI		*p*
			Lower	Upper				Lower	Upper	
Age	0.058	1.060	0.994	1.130	0.074	0.000	1.000	0.932	1.073	0.997
Male sex	−0.715	0.489	0.120	2.002	0.320	0.120	1.128	0.254	5.010	0.875
EBV DNA ≥ ULN (copies/mL)	1.905	6.716	1.379	32.709	0.018	1.316	3.728	0.721	19.281	0.117
PIV	0.001	1.001	1.001	1.002	<0.001					
PIV > Cutoff						3.596	36.452	3.337	398.237	0.003

β, regression coefficient; HR, hazard ratio; CI, confidence interval; EBV, Epstein–Barr virus; ULN, upper limit of normal; PIV, pan-immune-inflammation value. *p* < 0.05 considered statistically significant. Model 1 includes PIV as a continuous variable; Model 2 includes PIV dichotomized according to the ROC-derived cutoff.

**Table 8:** Univariate cox regression analysis on progression-free survival.

	Univariate Cox Regression Analysis				
	β	HR	95% CI		*p*
			Lower	Upper	
Age	0.015	1.015	0.983	1.047	0.370
Male sex	1.068	2.908	0.670	12.634	0.154
EBV DNA ≥ ULN (copies/mL)	1.431	4.183	1.418	11.811	0.007
PIV	0.001	1.001	1.001	1.001	<0.001
PIV > Cutoff	2.055	7.808	2.866	21.273	<0.001
NLR	0.432	1.540	1.296	1.830	<0.001
NLR > Cutoff	1.786	5.967	2.352	15.141	<0.001
PLR	0.013	1.013	1.007	1.019	<0.001
PLR > Cutoff	1.536	4.644	1.739	12.404	0.002
SII	0.001	1.001	1.001	1.002	<0.001
SII > Cutoff	1.382	3.982	1.604	9.881	0.003
HALP	−0.085	0.919	0.879	0.961	<0.001
HALP < Cutoff	1.836	6.271	2.342	16.790	<0.001
Induction + CCRT (vs. definitive CCRT)	−0.325	0.722	0.239	2.179	0.564
Stage 3–4 A (vs. stage II)	−0.205	0.815	0.270	2.460	0.716
Presence of comorbidity	0.525	1.691	0.677	4.223	0.261

β, regression coefficient; HR, hazard ratio; CI, confidence interval; EBV, Epstein–Barr virus; ULN, upper limit of normal; PIV, pan-immune-inflammation value; NLR, neutrophil-to-lymphocyte ratio; PLR, platelet-to-lymphocyte ratio; SII, systemic immune-inflammation index; HALP, hemoglobin–albumin–lymphocyte–platelet score; CCRT, concurrent chemoradiotherapy. *p* < 0.05 considered statistically significant.

**Table 9:** Multivariate cox regression analysis on progression-free survival.

	Multivariate Cox Regression Analysis Model 1 (Continuous PIV)					Multivariate Cox Regression Analysis Model 2 (PIV Dichotomized by Cutoff)				
	β	HR	95% CI		*p*	β	HR	95% CI		*p*
			Lower	Upper				Lower	Upper	
Age	−0.004	0.996	0.959	1.033	0.819	−0.020	0.980	0.945	1.016	0.980
Male sex	0.855	2.350	0.523	10.554	0.265	1.028	2.797	0.624	12.541	0.179
EBV DNA ≥ ULN (copies/mL)	1.261	3.528	1.187	10.486	0.023	0.992	2.696	0.853	8.519	0.091
PIV	0.001	1.001	1.000	1.001	<0.001					
PIV > Cutoff						2.254	9.525	2.838	31.972	<0.001

β, regression coefficient; HR, hazard ratio; CI, confidence interval; EBV, Epstein–Barr virus; ULN, upper limit of normal; PIV, pan-immune-inflammation value. *p* < 0.05 considered statistically significant. Model 1 includes PIV as a continuous variable; Model 2 includes PIV dichotomized according to the ROC-derived cutoff.

## Data Availability

The data supporting the findings of this study are available from the corresponding author upon reasonable request. The data are not publicly available due to privacy and ethical restrictions related to patient confidentiality.

## Abbreviations

AJCC	American Joint Committee on Cancer
AUC	Area under the curve
CBC	Complete blood count
CCRT	Concurrent chemoradiotherapy
CI	Confidence interval
DNA	Deoxyribonucleic acid
EBV	Epstein–Barr virus
GLOBOCAN	Global Cancer Observatory
HALP	Hemoglobin, albumin, lymphocyte, and platelet index
HR	Hazard ratio
IQR	Interquartile range
NLR	Neutrophil-to-lymphocyte ratio
NPC	Nasopharyngeal carcinoma
OS	Overall survival
PCR	Polymerase chain reaction
PFS	Progression-free survival
PIV	Pan-Immune-Inflammation Value
PLR	Platelet-to-lymphocyte ratio
ROC	Receiver operating characteristic
SII	Systemic immune-inflammation index
SPSS	Statistical Package for the Social Sciences
TNM	Tumor–node–metastasis
UICC	Union for International Cancer Control
ULN	Upper limit of normal
VIF	Variance inflation factor
